# The development and the psychometric evaluation of the Adolescents Intentions towards the Birth Options Scale in Greek

**DOI:** 10.18332/ejm/145968

**Published:** 2022-03-07

**Authors:** Dimitra Varnakioti, Kleanthi Gourounti, Antigoni Sarantaki, Chara Tzavara, Aikaterini Lykeridou

**Affiliations:** 1 Department of Midwifery, School of Health and Care Sciences, University of West Attica, Athens, Greece; 2 Centre for Health Services Research, Department of Hygiene, Epidemiology and Medical Statistics, Medical School, National and Kapodistrian University of Athens, Athens, Greece

**Keywords:** adolescents, birth options, planned behavior theory

## Abstract

**INTRODUCTION:**

Worldwide, the rising of caesarean section rates is a major public health issue. Little is known regarding birth attitudes held by students who are the next generation of parents. The aim of this study was to develop and assess the psychometric properties of the Adolescents Intentions towards Birth Options Scale (AIBOS), a self-report and short instrument assessing intentions towards birth options in young adolescents.

**METHODS:**

The AIBOS was framed by Ajzen’s theory of planned behavior and developed in a three-phase process using an integrated mixed-methods approach that included literature reviews, professional focus groups, and a psychometric survey evaluation. The psychometric evaluation was conducted by recruiting a sample of 480 high school students. Content validity, exploratory factor analysis, discriminant and construct validity, test-retest reliability and internal consistency were explored.

**RESULTS:**

The expert panel determined that the content validity was satisfactory. The final 17-item scale consisted of five factors explaining 48.9% of the total variance in the data. Discriminant validity was satisfactory. Cronbach’s α coefficient was over 0.7 for each factor, indicating acceptable internal consistency of the questionnaire. There was significant agreement in all subscales as emerged from test-retest.

**CONCLUSIONS:**

The AIBOS demonstrated good content validity, an easily interpretable five-factor structure, acceptable internal consistency, high test-retest reliability, and satisfactory discriminant and construct validity with sample characteristics. It is an easily comprehensible, easily completed tool, which matches the culture of young adolescents.

## INTRODUCTION

It has been more than thirty-five years since 1985 when the international healthcare community considered that the ideal rate for caesarean sections has to be between 10% and 15%^1^. In accordance with this, recent studies indicate that CS rates higher than 10% are not associated with reductions in maternal and newborn mortality rates^2^. Α caesarean section is a lifesaving procedure; however, it is associated with both maternal and perinatal risks while there is no evidence showing any benefits of the procedure for women or infants where it is not required^3^. However, in the last decades there has been an unprecedented and steady rise in CS rates in both developed and developing countries^4^. This could be explained through a complex multivariate analysis model; changes both regarding the distinguishing characteristics of the mother (increased percentage of elderly nulliparous women) and professional practice styles, personalized medicine instead of team obstetrical care, increased legal pressure for malpractice (defensive treatment), along with economic, organizational, social, and cultural factors^5-7^.

In Greece, the national rate has been climbing the last years, reaching 56.8% in 2016 or more in some areas^8^. A recent study indicates that in Greece, most women continue to give birth by CS, which leads to a major public health problem with economic, ethical and humanitarian implications^9^. In 2019, experts from the Organization of United Nations noted that in Greece the law about maternity protection was not being implemented uniformly and expressed concern about ongoing discrimination based on pregnancy and family responsibilities^10^. Many strategies have been introduced to reduce CS rates, including the continuing education of clinicians and communities regarding the benefits of vaginal birth and the risks of unnecessary CSs. While the investigation for interventional approaches is ongoing, worries have focused on the psychosocial and behavioral factors that affect and are affected by the dominance of caesarean delivery^11,12^.

Social cognitive theory models indicating that attitudes directly affect behaviors are currently being tested to predict preferred birth approaches^13-17^. Ajzen’s theory of planned behavior (TPB) has been successfully used to explain and predict behavior in a multitude of behavioral domains, from physical activity to drug use, from recycling to the choice of travel mode, from safer sex to consumer behavior, and from technology adoption to protection of privacy. According to this theory, human behavior is guided by three considerations: attitude toward the behavior, social pressure or subjective norm, and perceived behavioral control. A behavioral belief is the person’s subjective probability that performing a behavior of interest will lead to a particular outcome or provide a specific experience. Subjective norms refer to the expectation that others important to the person (e.g. friends, family, spouse, co-workers, one’s physician or supervisor) approve or disapprove the behavior under consideration or essential others themselves perform the behavior. Perceived behavior control refers to people’s beliefs that they can perform a given behavior. As a general rule, the more favorable the attitude and the subjective norm, and the greater the perceived control, the stronger should be the person’s intention to perform the behavior in question, which in our study is the choice of vaginal birth. Intention is thus assumed to be the immediate antecedent of behavior^17^.

Little is known regarding birth attitudes held by students who are the next generation of parents. Previous studies suggest that apart from the rise in the caesarean section rate, university students think about childbirth as a painful but normal process and report knowledge gaps regarding birth decisions^18-20^. Additional studies confirm that most young adults are misinformed regarding the common necessity of most childbirth interventions and the risks associated with performing those interventions when they are not medically necessary^21-25^. However, according to the author’s knowledge, there are no published studies on the birth attitudes of younger people, such as students of secondary education. Similarly, the authors were unable to identify any published studies regarding the development and use of tools measuring the birth attitudes of students. Therefore, this study aimed to develop and assess the psychometric properties of a self-report and short instrument assessing intentions towards birth options in young adolescents.

## METHODS

### Study design

This study was designed for scale development. The scale was prepared in the Greek language, and its development included three main phases: item generation, item reduction, initial validity testing (content validity testing), construct validity testing (exploratory factor analysis [EFA], reliability testing and criterion-related validity).

### Instrument development

*Phase I: Item generation*

The goal of phase I was to generate the items for the instrument from two main sources: a) an extensive literature review identifying data that examined attitudes towards birth decisions among men and women (pregnant and non-pregnant); and b) a focus group including six experts from different fields to evaluate the content validity index (CVI) of the developed questionnaire, a midwife, two university professors in midwifery, a PhD candidate from the University of West Attica, a high school educator and a clinical psychologist with a cognitive-behavioral background. Finally, a 19-item pool of items regarding attitudes and intentions on birth options in young populations was established. It is worth mentioning that the Childbirth Fear to Prior pregnancy scale (CFPP), assessing fear of childbirth prior to pregnancy, has been a significant guide to developing our instrument^26^.

*Phase II: Content validity testing*

Once the item pool was developed from phase I, the goals of phase II were to assess the content validity and reduce the number of questions for further scale development by assembling the panel of experts. The 19 items that were kept were the clearest and the most concise.

The content validity was assessed by determining the face validity and the content validity index (CVI). To evaluate whether items were relevant, clear, and essential, experts were given a critical appraisal sheet with the following four inquiries: 1) the relevance of each question in the tool (how important the question is); 2) the clarity of each question (how clear the wording is); 3) the essentiality of each question (how necessary the question is); and 4) recommendations for improvement of each question (reference). The experts were asked to rate each item on a 4-point scale ranging from 1 to 4 (1 = not relevant, 2 = somewhat relevant, 3 = quite relevant, and 4 = highly relevant) according to the applicability of the expression and content to the local culture and the research objective. Ratings of 1 and 2 are considered content invalid, while ratings of 3 and 4 are considered content valid. The CVI of each item (I-CVI) was calculated as the ratio of the number of ‘quite relevant’ and ‘highly relevant’ expert opinion responses to the number of experts. The overall CVI of the questionnaire was calculated as the average of the I-CVIs of all items. Items with an I-CVI lower than 0.78 were considered candidates for revision, and items with very low values were candidates for deletion. A CVI rating greater than 0.8 represented satisfactory content validity^27,28^. The experts suggested revision in two questions to match with the culture and the background of young adolescents. The majority of items were considered relevant, with an I-CVI over 0.78.

*Phase III: Reliability and validity testing*

The goals of the phase IIΙ were to assess validity, explore the instrument’s factor structure and internal consistency by using Cronbach’s alpha coefficient, the tool’s stability, by evaluating the test-retest reliability via intraclass correlation coefficient (ICC), and the discriminant and construct validity.

### Sample

The study was conducted in fourteen vocational high schools in Greece. Data were collected between October 2019 and April 2021. All students of first grade were invited to participate. A total sample of 480 students consented to participate. Due to the specialty of the project, instructions were provided by the researchers in every school unit, and additional clarifications were available for each of the students during the questionnaire administration.

The test-retest reliability of the questionnaire was calculated for 30 students of the sample who agreed to repeat AIBOS two weeks after the first administration.

### Instruments

The version of the AIBOS that emerged from phases I, II, and III, of the instrument development process consisted of 19 items designed to access the constructs of the theory of planned behavior. The questionnaire is a multidimensional instrument assessing the three main dimensions of the theory of planned behavior (attitudes, subjective norms and perceived behavioral control) by five factors: ‘Cognitive attitude towards vaginal birth’, ‘Cognitive attitude towards caesarean section’, ‘Affective attitude’, ‘Subjective norms’, and ‘Perceived behavioral control’. The ‘Cognitive attitude towards vaginal birth’ factor was measured by six items, ‘Cognitive attitude towards caesarean section’ factor was measured by four items and the ‘Affective attitude’ factor was measured by three items. ‘Subjective norms’ factor was measured by 2 items and ‘perceived behavioral control’ factor was measured by 2 questions. Every question was answered by a 5-item Likert scale ranging from ‘strongly disagree=1’ to ‘strongly agree=5’. Lower scores indicated negative attitudes and higher scores indicated positive attitudes.

Sociodemographic characteristics, sources of information regarding giving birth and educational needs were also examined. Permission for the use of the entire AIBOS can be obtained from the corresponding author at the request of professionals or organizations who wish to use it.

### Data analysis

Quantitative variables were expressed as means with standard deviation (SD), and absolute and relative frequencies. Exploratory factor analysis was carried out to evaluate construct validity, disclose underlying structures and reduce the number of items. Principal component analysis (PCA) was chosen as an extraction method using varimax rotation. Kaiser-Meyer-Olkin procedure for measuring sample adequacy was applied. The cut-off point for factor loadings was 0.40 and for eigenvalues was 1.00. Internal consistency reliability was determined by the calculation of Cronbach’s α coefficient. Factors with a coefficient ≥0.70 were considered acceptable. Intraclass correlation coefficient (ICC) was used to assess the reliability of the questionnaire, from test-retest procedure. Discriminant construct validity was evaluated by analyzing the association between the factors of the questionnaire and gender, field of study, and preference for a specific type of labor using Student’s t-tests. Statistical significance was set at p<0.05 and analyses were conducted using SPSS statistical software (version 24.0).

## RESULTS

### Sample characteristics

The sample consisted of 480 teenagers, aged 14–17 years with a mean age of 15.5 years (SD: 0.6). Sample’s characteristics are presented in Table 1. More than half (54.2%) of the teenagers were girls. Almost all teenagers (97.7%) were born in Greece. Most of the teenagers had parents who had completed high school. Moreover, 55.8% of the teenagers had chosen to study health sciences. Also, 89.0% of the teenagers would like to have children and 78.5 % would prefer a vaginal labor for them or their partner.

**Table 1:** Sample characteristics, Greece (N=480)

*Characteristics*	*n (%)*
**Age**, mean (SD)	15.5 (0.6)
**Gender**	
Boys	209 (43.5)
Girls	260 (54.2)
Prefer not to say	11 (2.3)
**Born in Greece**	469 (97.7)
**Father’s highest level of education**	
Primary school	69 (14.4)
High school	268 (55.8)
Technical School/Vocational Training Institute (VTI)	78 (16.3)
University degree	53 (11)
Post-graduate degree	8 (1.7)
PhD	4 (0.8)
**Mother’s highest level of education**	
Primary school	44 (9.2)
High school	251 (52.3)
Technical School/Vocational Training Institute (VTI)	92 (19.2)
University degree	74 (15.4)
Post-graduate degree	14 (2.9)
PhD	5 (1.0)
**Field of study**	
Agriculture and Environment	8 (1.7)
Administration and Economics	42 (8.8)
Structured Environment and Architectural Design	9 (1.9)
Applied Arts	15 (3.2)
Electronics and Automation	19 (4.0)
Engineering	55 (11.6)
Shipping and Maritime Studies	16 (3.4)
Computing studies	46 (9.7)
Health sciences	265 (55.8)
**Can you imagine yourself having children somewhere in the future?**	
Yes	427 (89.0)
No	53 (11.0)
**Assuming you could choose the type of birth for your baby, what would you prefer it to be?**	
Vaginal birth	377 (78.5)
Caesarean section delivery	103 (21.5)

Teenagers’ knowledge and attitude towards vaginal labor are presented in Table 2. Most participants strongly agreed with the statements ‘I believe that people significant for me (family, relatives) would prefer my delivery (my partner’s delivery) to be vaginal’ and ‘I believe that healthcare providers (obstetricians, midwives) would prefer my delivery (my partner’s delivery) to be vaginal’, 61.3% and 52.9%, respectively. Only, 2.3% of the sample strongly agreed with the statement ‘I see vaginal birth as an outdated method of childbirth’ and 2.5% with the statement ‘Caesarean section is safer for the mother’.

**Table 2:** Participants’ answers, in each statement separately (N=480)

	*Strongly disagree n (%)*	*Disagree n (%)*	*Neither agree nor disagree n (%)*	*Agree n (%)*	*Strongly agree n (%)*
Normal or vaginal birth is a completely natural process that occurs at the end of a pregnancy and the baby is delivered vaginally	6 (1.3)	15 (3.1)	87 (18.1)	241 (50.2)	131 (27.3)
Women give birth mainly by normal childbirth and caesarean sections in a population should not be more than a small percentage of all births	18 (3.8)	57 (11.9)	191 (39.8)	168 (35.0)	46 (9.6)
In Greece most women give birth with vaginal delivery	26 (5.4)	87 (18.1)	176 (36.7)	158 (32.9)	33 (6.9)
Vaginal birth is safer for the mother	11 (2.3)	57 (11.9)	119 (24.8)	178 (37.1)	115 (24)
Vaginal birth is safer for the baby	11 (2.3)	48 (10.0)	165 (34.4)	170 (35.4)	86 (17.9)
After a vaginal birth the recovery time is faster and the postpartum pain less	16 (3.3)	44 (9.2)	164 (34.2)	170 (35.4)	86 (17.9)
Normal childbirth activates the early bonding of mother–newborn and increases the success of breastfeeding	9 (1.9)	35 (7.3)	207 (43.1)	164 (34.2)	65 (13.5)
A caesarean section delivery is a surgical procedure required only for medically indicated reasons (the safety of the mother or the baby)	23 (4.8)	84 (17.5)	91 (19.0)	170 (35.4)	112 (23.3)
Caesarean section could be seen as a means to avoid the pain of a vaginal birth	34 (7.1)	95 (19.8)	149 (31)	160 (33.3)	42 (8.8)
Caesarean section is safer for the mother	57 (11.9)	124 (25.8)	215 (44.8)	72 (15)	12 (2.5)
Caesarean section is safer for the baby	45 (9.4)	95 (19.8)	233 (48.6)	92 (19.2)	14 (2.9)
Caesarean section could be suggested to provide the convenience to schedule the time of childbirth	23 (4.8)	43 (9.0)	233 (48.5)	141 (29.4)	40 (8.3)
In Greece most women give birth with caesarean section delivery	15 (3.1)	125 (26.0)	196 (40.8)	111 (23.1)	33 (6.9)
Vaginal birth is generally unpredictable	11 (2.3)	44 (9.2)	122 (25.4)	216 (45)	87 (18.1)
I see vaginal birth as an outdated method of childbirth	123 (25.6)	160 (33.3)	123 (25.6)	63 (13.1)	11 (2.3)
I believe that people significant for me (family, relatives) would prefer my delivery (my partner’s delivery) to be vaginal	53 (11.0)	28 (5.8)	8 (1.7)	97 (20.2)	294 (61.3)
I believe that healthcare providers (obstetricians, midwives) would prefer my delivery (my partner’s delivery) to be vaginal	86 (17.9)	23 (4.8)	11 (2.3)	106 (22.1)	254 (52.9)
I think that I (my partner) will have self-control and will confidently handle the process and the pain of a childbirth	34 (7.1)	67 (14.0)	161 (33.5)	151 (31.5)	67 (14)
I think that labor pain will be too intense. I am afraid that I (my partner) might panic and not know what to do during labor and birth	32 (6.7)	113 (23.5)	156 (32.5)	121 (25.2)	58 (12.1)

### Factor structure of the AIBOS

The results from the exploratory factor analysis are presented in Table 3. KMO value was 0.7 and Bartlett’s test of sphericity was statistically significant, (χ^2^=, df=, p<0.001). The proposed five factors were finally confirmed by the emerging factor analysis. All factors combined explained 48.9% of the variance. Factor ‘Cognitive attitude towards vaginal labor’ had 6 items and explained 13.1% of the variance. Factor ‘Cognitive attitude towards caesarean section’ had 4 items and explained 10.6% of the variance. Factor ‘Affective attitude’ had 3 items and explained 9.1% of the variance. Factors ‘Subjective norms’ and ‘Perceived behavior control’ had 2 items each and explained 8.4% and 7.7% of the variance, respectively. Two items (‘In Greece, most women give birth with vaginal delivery’ and ‘In Greece, most women give birth with caesarean section delivery’) initially attributed to the cognitive attitude factors had loadings lower than 0.4, thus were deleted and were not included in these factors. Therefore, the instrument finally consisted of 17 items. Cronbach’s α coefficient was >0.7 for each factor, indicating acceptable internal consistency of the questionnaire. The overall Cronbach α coefficient was 0.76.

**Table 3:** Loadings from exploratory factor analysis with principal components method, after varimax rotation (N=480)

		*Knowledge on vaginal labor*	*Knowledge on caesarean section*	*Affective attitude*	*Subjective norms*	*Perceived behavior control*
1	Normal or vaginal birth is a completely natural process that occurs at the end of a pregnancy and the baby is delivered vaginally			0.59		
2	Women give birth mainly by normal childbirth and caesarean sections in a population should not be more than a small percentage of all births	0.64				
3	In Greece most women give birth with vaginal delivery					
4	Vaginal birth is safer for the mother	0.72				
5	Vaginal birth is safer for the baby	0.71				
6	After a vaginal birth the recovery time is faster and the postpartum painless	0.44				
7	Normal childbirth activates the early bonding of mother–newborn and increases the success of breastfeeding	0.40				
8	A caesarean section delivery is a surgical procedure required only for medically indicated reasons (the safety of the mother or the baby)	0.45				
9	Caesarean section could be seen as a means to avoid the pain of a vaginal birth		0.53			
10	Caesarean section is safer for the mother		0.72			
11	Caesarean section is safer for the baby		0.71			
12	Caesarean section could be suggested to provide the convenience to schedule the time of childbirth		0.40			
13	In Greece most women give birth with caesarean section delivery					
14	Vaginal birth is generally unpredictable			0.60		
15	I see vaginal birth as an outdated method of childbirth			-0.48		
16	I believe that people significant for me (family, relatives) would prefer my delivery (my partner’s delivery) to be vaginal				0.42	
17	I believe that healthcare providers (obstetricians, midwives) would prefer my delivery (my partner’s delivery) to be vaginal				0.41	
18	I think that I (my partner) will have self-control and will confidently handle the process and the pain of a childbirth					-0.81
19	I think that labor pain will be too intense. I am afraid that I (my partner) might panic and not know what to do during labor and birth					0.84
% Variance explained		13.1	10.6	9.1	8.4	7.7
Cronbach’s α		0.75	0.75	0.71	0.78	0.77
Mean (SD)		3.53 (0.61)	3.00 (0.64)	3.78 (0.63)	4.01 (1.13)	2.91 (0.92)

### Test-retest reliability

Test-retest results are presented in Table 4. There was significant agreement in all factors as emerged from test-retest.

**Table 4:** Intraclass correlation coefficients (ICC) from test-retest

	*ICC*	*p*
Knowledge on vaginal labor	0.85	<0.001
Knowledge on caesarean section	0.82	<0.001
Affective attitude	0.90	<0.001
Significant others	0.89	<0.001
Perceived behavior control	0.87	<0.001

### Discriminant construct validity

To access the discriminant validity of the questionnaire, demographic variables (gender) and students’ future preferences (field of studies, mode of delivery) were associated with instrument factors. It was found that girls had significantly greater mean scores in all factors, except of ‘Knowledge on caesarean section’, compared to boys. Teenagers who would study health sciences in the future had significantly greater scores in ‘Knowledge on vaginal labor’, ‘Affective attitude’, ‘Significant others’ and significantly lower scores in ‘Knowledge on caesarean section’ comparison to teenagers who would study non-medical sciences. Moreover, teenagers who would prefer to have a vaginal birth for themselves or their partner had significantly greater scores in ‘Knowledge on vaginal labor’, ‘Affective attitude’, ‘Significant others’, ‘Perceived behavior control’ and significantly lower scores in ‘Knowledge on caesarean section’ in comparison to teenagers who would prefer to have a caesarean section. Associations between participants’ gender, field of study and selected type of labor are given in the Supplementary file.

## DISCUSSION

Birth-related attitudes and beliefs have been constantly examined during pregnancy, however availability of data regarding adolescents’ attitudes towards birth options are rather scarce.

Studies propose strategies that involve eliminating prejudice towards vaginal childbirth through raising awareness regarding the side effects of CS and the benefits of vaginal birth. Moreover, strategies that promote women’s confidence towards their ability to deliver naturally would potentially reduce unnecessary CS rates^29-33^. However, high rates of CS could suggest that these interventions may not be either consistently or timely implemented.

Recent research suggests for the immediate commencement of interventions aimed at reducing CS rates before parenthood, as birth stances are formed prior to pregnancy and are potentially influenced by modifiable factors such as fear of childbirth and lack of knowledge regarding birth decisions^34-36^.

The purpose of this study was to develop and validate a self-report measure of intentions towards birth options among young adolescents via the various components of the TPB. Consequently, the questionnaire was developed to examine adolescent’s attitudes towards vaginal birth and caesarean section (knowledge, beliefs, feelings), adolescent’s perceptions of significant others’ beliefs about birth options, adolescent’s perceived behavior control and intentions regarding birth options. Therefore, the questionnaire was named Adolescent’s Intentions towards Birth Options Scale (AIBOS)

Through an iterative, rigorous instrument development process, the AIBO scale was developed and tested. The AIBOS was developed by using an integrated mixed methods approach that included literature reviews, professional focus groups, expert consultations, and a psychometric survey evaluation. The AIBO scale demonstrated good content validity, an easily interpretable five-factor structure, acceptable internal consistency and test-retest reliability, and satisfactory discriminant validity with sample characteristics.

The results of the exploratory factor analysis suggest that discriminative capacity existed among the items and that a five-factor solution was the most appropriate. The factors were interpreted and labelled: ‘cognitive component of vaginal birth’, ‘cognitive component of caesarean section’, ‘affective component of vaginal birth’, ‘subjective norms-significant others’ and ‘perceived behavior control’, in accordance with the TPB.

The five-factor solution of the AIBO 17-item scale cumulatively accounted for 48.9% of the variance. Convergent validity (e.g. the extent to which a test correlates with other variables with which it theoretically should correlate) could not be assessed because, and according to our knowledge, no other validated instrument measuring attitudes to birth options in adolescents was available.

Known groups method testing showed that girls had significantly greater scores in all factors, except for ‘Knowledge on caesarean section’, compared to boys. Teenagers who would study health sciences had significantly greater scores in ‘Knowledge on vaginal labor’, ‘Affective attitude’, ‘Significant others’ and significantly lower scores in ‘Knowledge on caesarean section’. Moreover, teenagers who would prefer a vaginal labor for them or their partner had significantly greater scores in ‘Knowledge on vaginal labor’, ‘Affective attitude’, ‘Significant others’, ‘Perceived behavior control’, and significantly lower scores in ‘Knowledge on caesarean section’.

### Limitations

Results of this study need to be interpreted within the light of some limitations. First, convenience sampling was used, although that was not planned. The research protocol had anticipated the participation of schools from various geographically located departments; however, the emergence of COVID-19 pandemic made all school communities rather skeptical of all live activities. Additionally, the sample of students was drawn only from vocational high schools due to the limitations of the Institute of Educational Policy (I.E.P) that suggests any surveys need to be integrated in relative courses. It is worth mentioning that ideally the researchers would have liked to collect data about the emotional state of the participants, but was prevented by a number of limitations due to the low average age of the sample. Thus, the results of this study may have introduced a selection bias and produced a non-representative sample of students, therefor they are not likely to be generalizable. It is essential to explore the psychometric properties and assess this scale among students from different settings (e.g. general high schools) and different geographical regions.

## CONCLUSIONS

The AIBOS was found to have satisfactory psychometric properties with a meaningful five-factor structure, good internal reliability and good discriminant and construct validity. It is an easily comprehensible, easily completed scale, which matches with the culture and the lifestyle of young adolescents. Since a wide range of non-clinical interventions are intended to reduce unnecessary caesarean section births, targeting evaluation towards various stakeholders (women or families, healthcare professionals, healthcare organizations or facilities), AIBOS may be used for evaluating intentions towards birth options long before parenthood starts. Future research should investigate the factorial structure of the AIBOS and must be verified in another sample of students through a confirmatory factor analysis.

## Data Availability

The data supporting this research are available from the authors on reasonable request.
